# The Semaphorin 3A Inhibitor SM-345431 Accelerates Peripheral Nerve Regeneration and Sensitivity in a Murine Corneal Transplantation Model

**DOI:** 10.1371/journal.pone.0047716

**Published:** 2012-11-09

**Authors:** Masahiro Omoto, Satoru Yoshida, Hideyuki Miyashita, Tetsuya Kawakita, Kenji Yoshida, Akiyoshi Kishino, Toru Kimura, Shinsuke Shibata, Kazuo Tsubota, Hideyuki Okano, Shigeto Shimmura

**Affiliations:** 1 Department of Ophthalmology, Keio University School of Medicine, Tokyo, Japan; 2 Genomic Science Laboratories, Dainippon Sumitomo Pharma Co., Ltd., Tokyo, Japan; 3 Department of Physiology, Keio University School of Medicine, Tokyo, Japan; University of Iowa, United States of America

## Abstract

**Background:**

Peripheral nerve damage of the cornea is a complication following surgery or infection which may lead to decreased visual function. We examined the efficacy of the semaphorin 3A inhibitor, SM-345431, in promoting regeneration of peripheral nerves in a mouse corneal transplantation model.

**Methodology/Principal Findings:**

P0-Cre/Floxed-EGFP mice which express EGFP in peripheral nerves cells were used as recipients of corneal transplantation with syngeneic wild-type mouse cornea donors. SM-345431 was administered subconjunctivally every 2 days while control mice received vehicle only. Mice were followed for 3 weeks and the length of regenerating nerves was measured by EGFP fluorescence and immunohistochemistry against βIII tubulin. Cornea sensitivity was also measured by the Cochet-Bonnet esthesiometer. CD31 staining was used to determine corneal neovascularization as a possible side effect of SM-345431. Regeneration of βIII tubulin positive peripheral nerves was significantly higher in SM-345431 treated mice compared to control. Furthermore, corneal sensitivity significantly improved in the SM-345431 group by 3 weeks after transplantation. Neovascularization was limited to the peripheral cornea with no difference between SM-345431 group and control.

**Conclusions/Significance:**

Subconjunctival injections of SM-345431 promoted a robust network of regenerating nerves as well as functional recovery of corneal sensation in a mouse keratoplasty model, suggesting a novel therapeutic strategy for treating neurotrophic corneal disease.

## Introduction

The cornea is a densely innervated tissue with abundant sensory and autonomic nerve fibers involved in the homeostasis of the ocular surface. Numerous studies have shown that soluble factors released by nerves are vital for maintenance and wound healing of the corneal epithelium (See review by Müller et al. [1]). However, less is known about regeneration of nerve fibers themselves following trauma or surgery. Surgery involving incisions to the cornea include corneal transplantation and refractive surgery, which cause a delay in wound healing and dry eye in a subset of patients [2]–[5]. Treatment strategies for these patients mainly focus on supplementing neurotrophic factors such as substance P and insulin-like growth factor 1 to the ocular surface [6], [7]. Although such attempts show promising results, developing a way to promote nerve regeneration would be a more ideal approach to treating peripheral nerve damage.

Nerve growth during development is determined by guidance molecules in the embryo that provide both attractive and repulsive signals to the advancing axons. Major guidance molecules in the embryo include netrins, semaphorins and ephrins that can enhance or inhibit neuronal growth depending on the stage of development [8], [9]. Some of these factors, such as Nogo-A and myelin-associated glycoprotein are also expressed in the postnatal and adult nervous system where they are believed to play a role in nerve regeneration [10]–[12]. Another such chemorepellent found in adult tissue is semaphorin 3A (Sema 3A), which is an extracellular matrix molecule that contributes to the inhibition of axonal regeneration [13], [14], and functions by binding with neuropilin-1 on growth cone filopodial tips [15]. During development, Sema3A is involved in nerve generation by acting as a negative regulator of nerve progression into the cornea of chick embryos [16], [17] Tanelian et al. demonstrated that forced expression of Sema3A in corneal epithelial cells in adult rabbits caused repulsion of A-delta and C fiber trigeminal sensory afferents *in vivo* [18]. Furthermore, Morishige et al. showed the presence of Sema3A in wing cells and basal cells of the adult rat corneal epithelium, stromal keratocytes and the corneal endothelium [19]. Recently, a selective inhibitor of Sema3A [20] was shown to enhance regenerative response and functional recovery in a spinal cord injury model [21]. We therefore hypothesized that a selective Sema3A inhibitor can be used to regenerate peripheral nerves in a mouse corneal transplantation model. In this study, we examined both anatomical regeneration of the nerve network by immunohistochemistry, as well as functional recovery of the blink reflex using an esthesiometer. We also examined the effects of the Sema3A inhibitor on corneal neovascularization since Sema3A is reported to inhibit VEGF-induced neovascularization [21], [22].

## Results

### Peripheral nerve damage following transplantation

**Figure 1:**
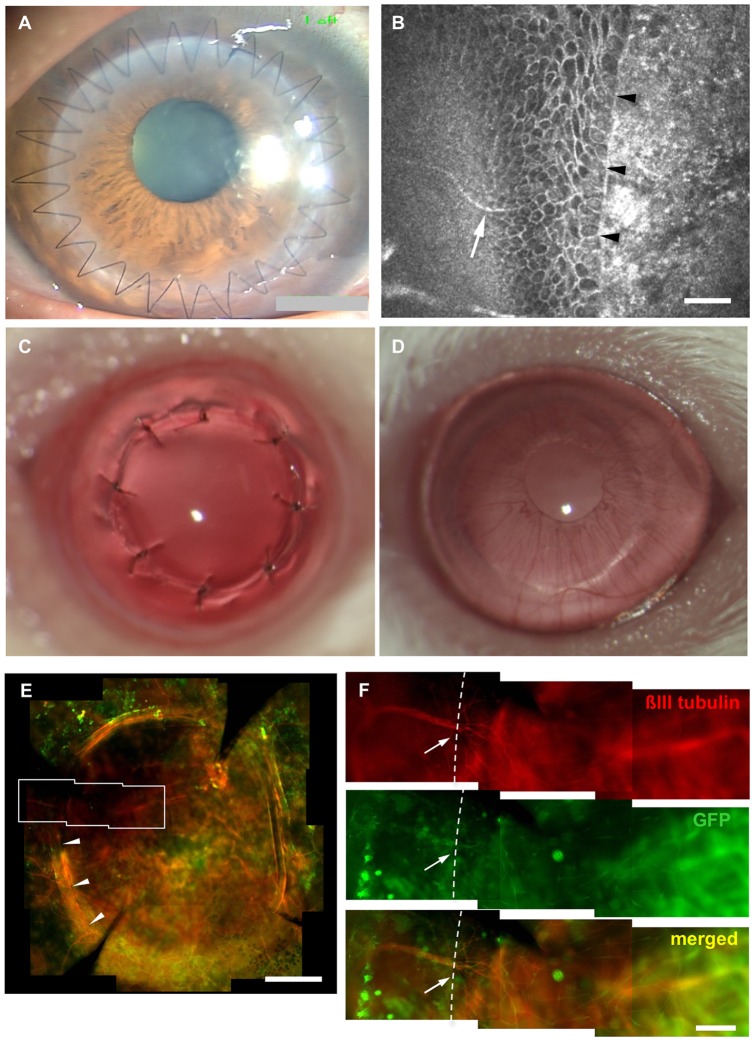
Peripheral nerve damage following corneal transplantation. Corneal transplantation in humans invovles a 360 degree full thickness incision of the cornea (A), and *in vivo* confocal micorscopy reveals recipient nerve fibers (white arrow) extending to the donor-host junction (arrow heads)(B). None of the 6 patients exmined demonstrated signs of nerve regerenation within the donor at 3 months. A murine transplantation model was developed by transplanting 2 mm wild-type donors into P0-Cre/Floxed-EGFP hosts (C) Sutures were removed after 7 days to avoid excessive inflammation (D). Peripheral nerves can be observed extending to the donor host juntion (arrow heads in E) by positive βIII tubulin staining and GFP in a magnified view (F). Dotted line in (F) shows the border of the donor and recipient cornea. Scale bar  = 50 µm in B, 500 µm in E and 100 µm in F.

Corneal transplantation involves a 360 degree, full-thickness excision of the recipient cornea followed by suturing of the donor cornea (Fig. 1A). *In vivo* confocal microscopy in transplanted patients showed nerve fibers extending from the periphery towards the host-donor margin, but not beyond the donor edge (Fig. 1B). None of the 6 patients examined showed evidence of regenerating nerves at 3 months following transplantation.

In animal experiments, corneal transplantation in P0-Cre/Floxed-GFP mice was done with 2 mm donor buttons using 8 sutures, which were removed 7 days following surgery to avoid suture-induced secondary inflammation and neovascularization (Fig. 1 C, D). Nerve fibers within flat mount sections of transplanted eyes can be observed by βIII tubulin staining overlapping with GFP fluorescence driven by the P0 promoter (Fig. 1 E). After 3 weeks, GFP positive nerve fibers can be observed regenerating from truncated nerve terminals into wild-type donor corneas, whose nerve do not express GFP (Fig. 1F).

### The Sema3A inhibitor SM-345431 enhances peripheral nerve regeneration

**Figure 2:**
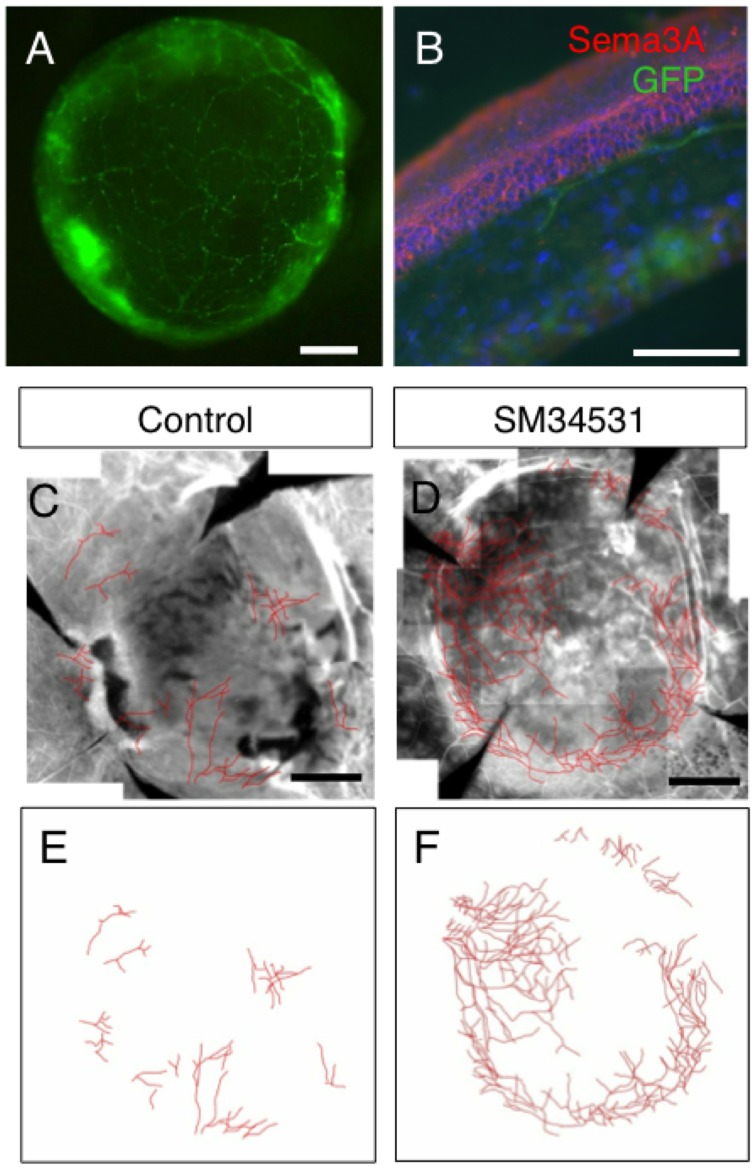
SM-345431 enhances nerve regeneration into the donor cornea. Peripheral nerves in the cornea of P0-Cre/Floxed-EGFP mice can be observed by GFP fluorescence (A), and immunohistochemisty of a 40 µm-thick frozen section shows the expression of Sema3A in the basal and suprabasal layers of the corneal epithelium (B, red) and GFP-positive major nerve fibers running through the corneal stromal layer under the epithelium (B, Green). Scale bar  = 500 µm in A, 100 µm in B. GFP and βIII tubulin double-positive nerve fibers extending into the donor cornea were traced on an image processing software. The SM-345431-treated group showed a more robust network of regenerating nerves (D, F) compared to vehicle control (C, E).

**Figure 3:**
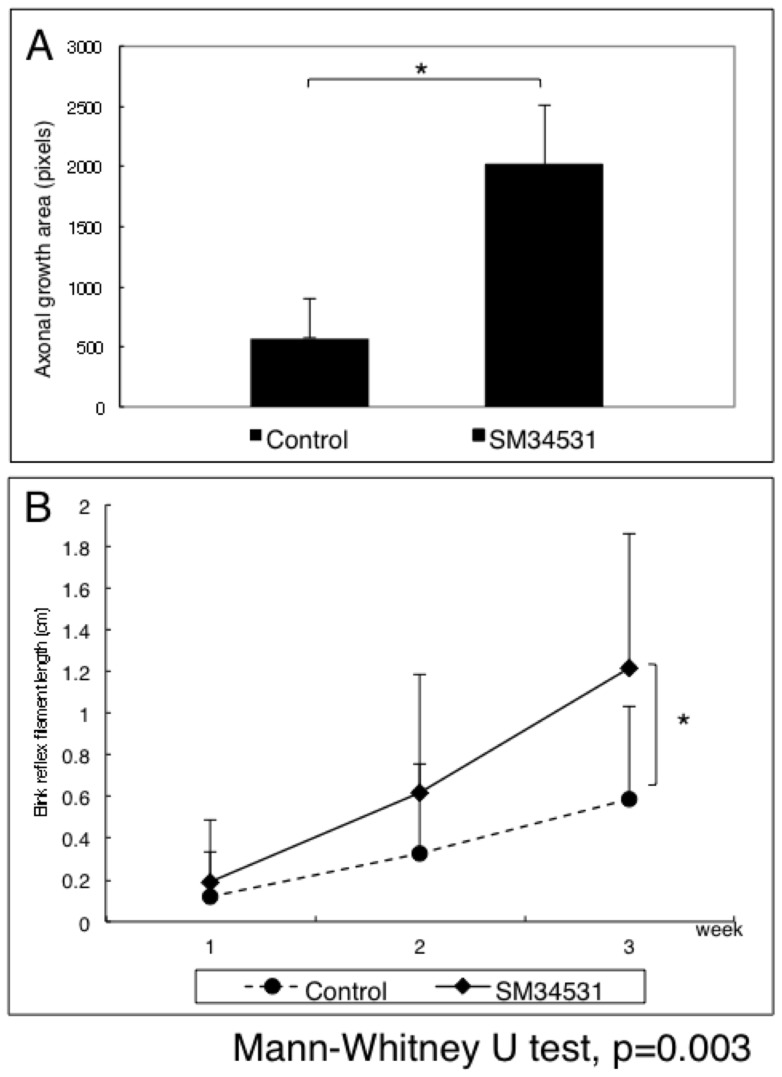
SM-345431 enhances the corneal blink reflex. The extension of nerve regeneration in the SM-345431 treated-group was significantly greater than control as measured by the total pixel count of traced axonal growth (A). In order to semi-quantitatively measure corneal sensation, a Cochet-Bonnet esthesiometer was used to measure the length of filament required to elicit a blink reflex. Three weeks postoperatively, the Sema3A inhibitor-treated group showed significant improvement in corneal sensitivity compared to the control group (B).

P0-Cre/Floxed-GFP mice used as transplant hosts show GFP positive peripheral nerves within the corneal stroma (Fig. 2A), while the basal and suprabasal epithelial cells express Sema3A (Fig. 2B). The corneal endothelium also expressed low levels of Sema3A. Line tracings of GFP + nerves 3 weeks following transplantation of wild type donor corneas show a robust network of nerves extending into the donor cornea in SM-345431-treated mice (Fig. 2 C, D). However, nerve regeneration was limited to the peripheral cornea in untreated mice (Fig. 2 E, F). Semi-quantitative analysis of regenerating nerve length showed significantly higher nerve growth in the SM-345431 treated group compared to untreated control (Fig. 3A). Furthermore, corneal sensitivity was compared using the Cochet-Bonnet esthesiometer which measures the amount of stimulation required for a blink reflex. By 3 weeks following transplantation, corneal sensitivity was higher in the SM-345431 treated group compared to untreated control (Fig. 3B). Post-operative treatment with SM-345431 promoted both peripheral nerve growth and recovery of corneal sensitivity in mice following complete excision of nerves by full-thickness corneal transplantation.

### Effect of SM-345431 on neovascularization

**Figure 4:**
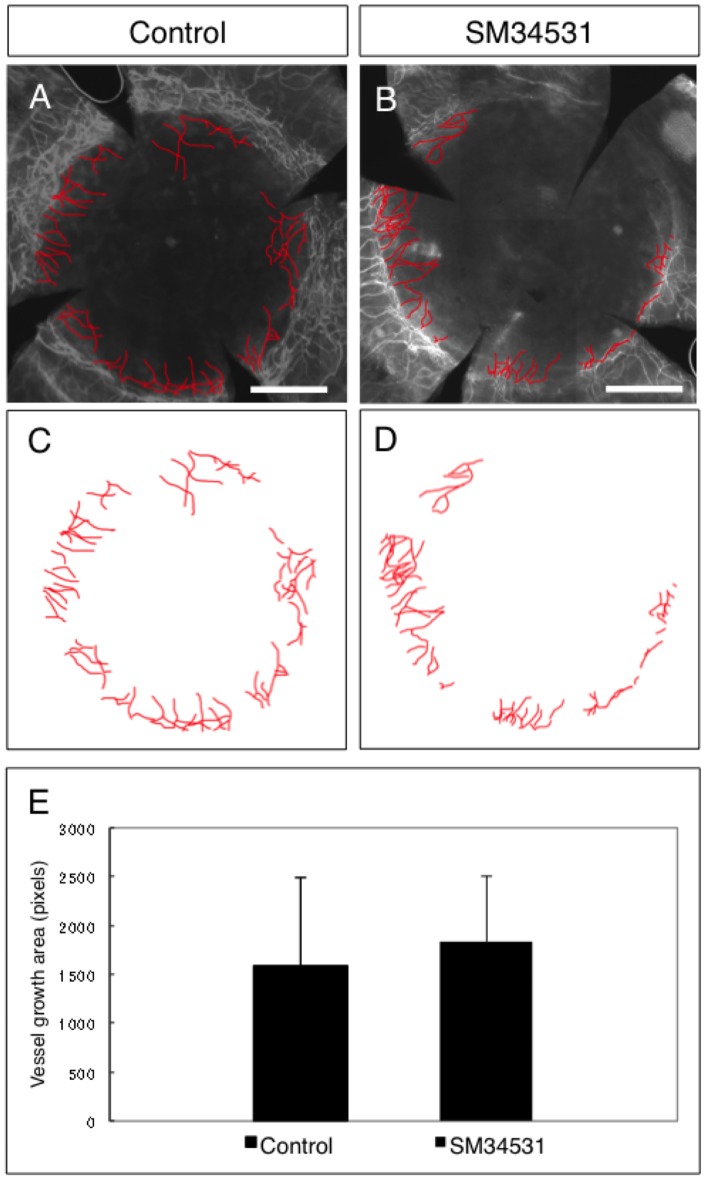
SM-345431 does not induce angiogenesis. Sema3A is also known to suppress VEGF-induced neovascularization. In order to examine possible angiogenesis by inhibiting Sema3A, neovascularization in the transplanted grafts were quantified by anti-CD31 immunostaining (A, B) and image tracing (C, D). There was no significant difference in vascular area between the two groups (E). Scale bar  = 500 µm in A, B.

Sema3A is known to inhibit VEGF-induced neovascularization by competing with VEGF for binding to their common receptor, neuropilin-1 (NP-1) [22]. Furthermore, SM-345431 acts directly on Sema3A to inhibit the binding of Sema3A to NP-1 (Cho et al., in preparation). Therefore, inhibition of Sema3A may induce vessel growth into the cornea. Since the cornea is an avascular tissue, neovascularization would be a deleterious side effect of SM-345431. We found that at least in doses used in our experiments, there was a slight increase in vessel growth within the donor cornea compared to control, however, the difference was not statistically significant (Fig. 4). Peripheral invasion of some new blood vessels were expected since sutures are often used as an inducer of neovascularization in the mouse cornea [23].

### Effect of SM-345431 on corneal epithelial cells

**Figure 5:**
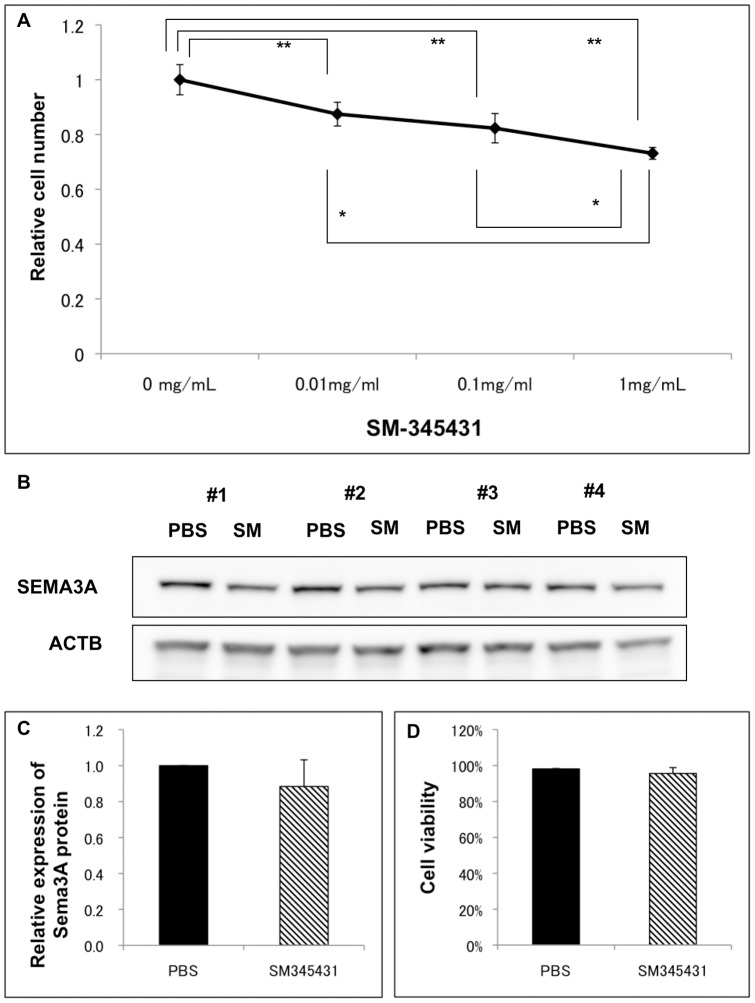
SM-345431 inhibits epithelial cell proliferation but does not affect cell viability or Sema3A production. Since the corneal epithelium was the predominant source of Sema3A in the cornea, the effect of SM-345431 on cultured murine corneal epithelial cells were observed. There was a slight dose-dependent inhibition on cell proliferation (A) (n = 5, *, **P<0.05, One way ANOVA followed with Scheffe's F test). However, there was no effect on Sema3A production observed by western blot (B, C) or on cell viability under the dose used in the *in vivo* experiments.

Since Sema3A was expressed predominantly by the corneal epithelium, we observed the effects of SM-345431 on cultured corneal epithelial cells using an established murine corneal epithelial cell line [24]. We found that SM-345431 showed a slight dose-dependent inhibition on cell proliferation (Fig. 5A), although no deleterious effects were observed in the *in vivo* experiments. There was no effect on Sema3A production by epithelial cells (Fig. 5 B, C) and cell viability was not affected by the dose of SM-345431 used in the study (Fig. 5D).

## Discussion

Sema3A is an extracellular matrix molecule that is expressed in the mouse cornea during development [25], as well as in the adult corneal in rats [19] and cultivated human corneal fibroblasts [26]. The role of Sema3A in the adult cornea is unknown, although reports have suggested a role in epithelial wound healing [27]. Cao et al. found a 10-fold increase in corneal Sema3A expression in a mouse wound healing model [28]. However, the most studied function of Sema3A is its effect on axonal growth during development and wound healing, and therefore, a similar role is to be expected in the cornea. We found that the selective Sema3A inhibitor SM-345431 enhanced nerve regeneration following corneal transplantation, which more importantly, was accompanied by recovery of corneal sensation. The clinical implications of this are large since there are no other methods to promote such recovery in the cornea, which is one of the most richly innervated tissues of the body.

Decreased corneal nerve sensation is associated with complications due to diabetes mellitus [29], herpes simplex viral infection [30] and trauma including surgical intervention. Neurotrophic ulcers due to decreased sensation in patients following corneal transplantation is a serious complication that can lead to corneal melting and eventual failed grafts [31]. Corneal sensation also plays a role in basal tear secretion [32]. Dry eye due to decreased tear secretion is a major complication after laser-assisted in situ keratomileusis (LASIK) surgery for myopia, where most of the superficial corneal nerve fibers are severed during surgery [2], [3]. Over one million cases of LASIK are performed every year in the United States alone, and tear secretion in these patients do not recover for up to one year following surgery [5]. The only therapeutic agents available for these patients are tear supplements and lubrication.

Previous attempts to treat hypoesthesia include supplementing growth factors and humoral factors associated with nerves such as substance P [6], [7]. While such attempts seem successful, regenerating nerve function would be ideal in terms of functional recovery. Hypersensitivity to pain, or abnormal pain sensation (allodynia) may be a complication of enhanced innervation by sensory nerves. This is an aspect of the drug that requires further investigation. However, an analogue of SM-345431 did not induce signs of allodynia in a spinal cord injury model [21].

Another possible undesirable effect of Sema3A inhibitors is the promotion of vessel growth into the clear cornea. Sema3A inhibits VEGF-induced angiogenesis *in vivo* [22], and therefore, the use of an inhibitor of Sema3A may cause vessels to invade the cornea. Fortunately, we found that vessel growth was limited to the periphery, with no significant difference compared with control mice. Avascularity of the cornea is regulated by several intrinsic factors such as endostatin, thrombospondins 1 and 2, angiostatin and matrix metalloproteinases (MMPs) [33]–[36]. Therefore, a redundant mechanism may exist to counteract any effects of SM-345431 on angiogenesis.

Selective regeneration of nerves may make SM-345431 an ideal drug for the treatment of cornea neurotrophic disease. While our experiments were done by injecting SM-345431 subconjunctivally, the next step would be to formulate a drug that can be applied topically in the form of eye drops or ointments. Drug delivery should not be a problem since neurotrophic corneas have delayed wound healing and impaired epithelial tight junctions often observed clinically by the diffusion of fluorescent diagnostic dyes into the subepithelial stroma. Concomitant use of neurotrophic factors in addition to SM-345431 may also enhance further axonal growth [37]. The cornea is also rich in chondrotin sulfate proteoglycans, another extracellular matrix molecule known to inhibit axonal regeneration [38]. The use of chondroitinase may also enhance the effects of SM-345431 [39]. Other neuro-regulatory molecules studied in other organs may also play a role in nerve regeneration in the cornea. Both netrin-4 and ephrins-A1 were reported to inhibit epithelial cell proliferation [40], [41], however, their role in nerve homeostasis remains to be evaluated. In conclusion, our study shows that Sema3A plays a role in the suppression of peripheral nerve regeneration in the cornea and that SM-345431 may be a novel therapeutic agent for treating neurotrophic corneal disease.

## Materials and Methods

### Preparation of SM-345431

SM-345431 (vinaxanthone), a small molecular Sema3A inhibitor was isolated from the cultured broth of a funbus *Penicillium* sp. Strain SPF-3059. The detail procedure for the fermentation and purification were described previously [42]. To confirm the inhibitory activity, the Sema3A-induced growth cone collapse assay was employed. The detail of the assay was described in the same report.

### *In Vivo* Laser Scanning Confocal Microscopy

*In vivo* laser scanning confocal microscopy (Rostock Corneal Software Version 1.2 of the Heidelberg Retina Tomograph II; RCM/HRT II; Heidelberg Engineering GmbH, Dossenheim, Germany) was performed on 6 patients of corneal transplantation preoperatively and 3 months postoperatively. After the administration of topical anesthesia with 0.4% oxybuprocaine, the subject's chin was placed in a chin rest. The objective of the microscope was an immersion lens covered by a polymethylmethacrylate cap (Tomo Cap; Heidelberg Engineering GmbH). Comfort gel (Bausch&Lomb, GmbH, Berlin, Germany) was used as a coupling agent between the applanating lens cap and the cornea. The laser source used in the HRTII/RCM is a diode laser with a wavelength of 670 nm. The laser confocal microscope provides images that represent a coronal section of the cornea of 400×400 µm, which is 160,000 µm^2^, at a selectable corneal depth and is separated from adjacent images by approximately 1 to 4 µm and lateral resolution of 1 µm/pixel. Digital images were stored on a computer workstation at 30 frames/second.

### Murine transplantation model

Transgenic mice expressing Cre recombinase under control of the P0 promoter (P0-Cre) [43] were mated with EGFP reporter mice (CAG-CATloxP/loxP-EGFP) [44] to obtain P0-Cre/Floxed-EGFP transgenic mice. Adult wild-type ICR mice were purchased from CLEA Japan. All mice were used at 8 to 12 weeks of age. Each mouse was anesthetized by intramuscular injection of a mixture of 3.75 mg ketamine and 0.75 mg xylazine before all surgical procedures. All aspects of animal care and treatment were carried out according to the guidelines of the experimental animal care committee of Keio University, School of Medicine.

Penetrating keratoplasty was performed on P0-Cre/Floxed-EGFP transgenic mice. Donor corneas 2 mm in diameter were excised from wild type ICR mice and placed in the same sized recipient bed, and secured with eight interrupted sutures (11-0 nylon). Sutures were removed at 7 days after grafting. The Sema3A inhibitor (0.1 mg/mL) was diluted with 0.4% betamethasone and administered every two days by subconjunctival injection. To the control group, only betamethasone without the inhibitor was administered. They were followed up for three weeks postoperatively by slit lamp microscopy.

### Immunohistochemistry for semaphorin3A

Eyes were frozen in OCT compound immediately after enucleation and were sectioned at a thickness of 5 mm. The frozen sections were air dried, fixed in 4% paraformaldehyde (PFA; Wako Ltd., Osaka, Japan) for 10 minutes, and then incubated in fixative (Morphosave; Ventana Medical Systems, Tucson, AZ) for 15 minutes. Blocking was performed with 10% normal donkey serum in phosphate-buffered saline (PBS) for 30 minutes. Sections were then incubated with Sema3A primary antibody (rabbit, Abcam, Cambridge, UK) for 1 hour at room temperature. Immunoreactivity of primary antibodies was visualized with secondary antibody conjugated with Cy3 (Chemicon International, Temecula, CA). After they were washed with PBS, the sections were mounted (Permafluor; Beckman Coulter Inc., Miami, FL). Images were observed by a microscope (Axio Imager; Carl Zeiss Inc., Oberkochen, Germany) equipped with a digital camera (Axiocam; Carl Zeiss).

### Quantification of corneal re-innervation and neovascularization

All mice were sacrificed 3 weeks after surgery. Whole corneas were fixed in 4% PFA for 30 minutes at room temperature. Corneas were excised under a surgical scope and washed with PBS. PFA-fixed corneas were permeabilized with 0.3% Triton X-100 (Sigma-Aldrich, St. Louis, MO). Blocking was achieved with 2-hour incubation in 10% normal donkey serum. Corneas were incubated with anti βIII-tubulin (rabbit, SIGMA) or anti CD31 (rat, Becton Dickinson, Franklin Lakes, NJ) primary antibodies for 2 hours at room temperature. After three washes in PBS, corneas were incubated in donkey anti-rabbit secondary antibody conjugated to Cy3 (Chemicon International) for 2 hours at room temperature. After four relaxing radial incisions were made, the corneas were coverslipped with mounting medium (Permafluor; Beckman Coulter) and imaged with a fluorescence microscope (Axio Imager; Carl Zeiss Inc., Oberkochen, Germany). The fields of view at the level of the sub-basal corneal nerve plexus or neovessels were photographed with both GFP and Cy3, or FITC filters respectively (Axiocam; Carl Zeiss). Nerve fibers and neovessels in a corneal graft were traced and the length was calculated with commercial software (Photoshop; Adobe Systems, San Jose, CA) by counting all the pixels of the traced line.

### Measurement of corneal sensitivity

Blink reflex was tested once a week postoperatively using Cochet-Bonnet esthesiometer (Handaya, Japan). Unanesthetized mice were held by the scruff of the neck under a dissecting microscope and touching the center of the corneal graft with a nylon filament.

### Cell proliferation and viability assay

A mouse corneal epithelial cell line (TKE2) was maintained in serum free low calcium medium (defined keratinocyte SFM, Gibco, Life Technologies Corporation, Carlsbad, CA) as described previously [24]. For the cell proliferation assay, cells (1×10^4^/well) were subcultured in 96 well plates. On the following day, cells were treated with 10-fold serial concentration of SM345431 (0.01–1.0 mg/mL) for 1 day. After rinsing two times with PBS, cells were incubated with medium containing WST-1 assay solution (Takara Bio Inc., Shiga, Japan) for 2 hours. Supernatants were moved to new wells, and absorbance at 450 nm with a reference wavelength at 620 nm was measured by micro-plate reader. For the cell viability assay, cells were cultured in 25 cm^2^ flasks until they reached 50% confluence, and treated with 0.1 mg/mL SM-345431 for 1 day. Cells were dissociated by enzyme treatment (TrypLE Express, Gibco) and dead cells were stained with trypan blue (final 0.25%). Number of viable cells and dead cells were counted by hematocytemeter.

### Western blot analysis

Semi-confluent TKE2 cells in 100 mm dish were treated with 0.1 mg/mL SM-345431 for 1 day. Medium containing the same amount of PBS (final 1%) was used as control. Cells were rinsed with PBS twice, and dissolved in lysis buffer (M-PER, Thermo Fisher Scientific, Waltham, MA) with protein inhibitor cocktail (final 1%, Thermo Fisher). Western blot analysis was performed by using standard protocols with primary antibodies for β actin (rabbit, Abcam) and Sema3A. Chemiluminescence intensity was measured using a CCD camera system (ImageQuant LAS 4000 mini, GE Healthcare, Piscataway, NJ) with analyzing software (ImageQuant TL, GE healthcare).

### Statistical Analysis

All results were expressed as mean ± SD. Values were processed for statistical analyses (Mann-Whitney U test, unpaired Student's t-test or one way ANOVA followed with Scheffe's F test) and differences were considered statistically significant at P<0.05.

## References

[1] Müller LJ, Marfurt CF, Kruse F, Tervo TM (2003) Corneal nerves: structure, contents and function. Exp Eye Res 76: 521–542. 12697417 10.1016/s0014-4835(03)00050-2

[2] Aras C, Ozdamar A, Bahcecioglu H, Karacorlu M, Sener B, et al (2000) Decreased tear secretion after laser in situ keratomileusis for high myopia. J Refract Surg 16: 362–364. 10832986 10.3928/1081-597X-20000501-10

[3] Benitez-del-Castillo JM, del Rio T, Iradier T, Hernandez JL, Castillo A, et al (2001) Decrease in tear secretion and corneal sensitivity after laser in situ keratomileusis. Cornea 20: 30–32. 11188999 10.1097/00003226-200101000-00005

[4] Mathers WD, Jester JV, Lemp MA (1988) Return of human corneal sensitivity after penetrating keratoplasty. Arch Ophthalmol 106: 210–211. 3277607 10.1001/archopht.1988.01060130220030

[5] Toda I, Asano-Kato N, Komai-Hori Y, Tsubota K (2001) Dry eye after laser in situ keratomileusis. Am J Ophthalmol 132: 1–7. 11438046 10.1016/s0002-9394(01)00959-x

[6] Chikama T, Fukuda K, Morishige N, Nishida T (1998) Treatment of neurotrophic keratopathy with substance-P-derived peptide (FGLM) and insulin-like growth factor I. Lancet. 351: 1783–1784. 10.1016/s0140-6736(98)24024-4 9635953

[7] Brown SM, Lamberts DW, Reid TW, Nishida T, Murphy CJ (1997) Neurotrophic and anhidrotic keratopathy treated with substance P and insulinlike growth factor 1. Arch Ophthalmol 115: 926–927. 9230840 10.1001/archopht.1997.01100160096021

[8] Chisholm A, Tessier-Lavigne M (1999) Conservation and divergence of axon guidance mechanisms. Curr Opin Neurobiol 9: 603–615. 10508749 10.1016/S0959-4388(99)00021-5

[9] Cook G, Tannahill D, Keynes R (1998) Axon guidance to and from choice points. Curr Opin Neurobiol 8: 64–72. 9568393 10.1016/s0959-4388(98)80009-3

[10] Chen MS, Huber AB, van der Haar ME, Frank M, Schnell L, et al (2000) Nogo-A is a myelin-associated neurite outgrowth inhibitor and an antigen for monoclonal antibody IN-1. Nature 403: 434–439. 10667796 10.1038/35000219

[11] Domeniconi M, Cao Z, Spencer T, Sivasankaran R, Wang K, et al (2002) Myelin-associated glycoprotein interacts with the Nogo66 receptor to inhibit neurite outgrowth. Neuron 35: 283–290. 12160746 10.1016/s0896-6273(02)00770-5

[12] Schwab ME, Kapfhammer JP, Bandtlow CE (1993) Inhibitors of neurite growth. Annu Rev Neurosci 16: 565–595. 7681638 10.1146/annurev.ne.16.030193.003025

[13] De Winter F, Oudega M, Lankhorst AJ, Hamers FP, Blits B, et al (2002) Injury-induced class 3 semaphorin expression in the rat spinal cord. Exp Neurol 175: 61–75. 12009760 10.1006/exnr.2002.7884

[14] Pasterkamp RJ, Giger RJ, Ruitenberg MJ, Holtmaat AJ, De Wit J, et al (1999) Expression of the gene encoding the chemorepellent semaphorin III is induced in the fibroblast component of neural scar tissue formed following injuries of adult but not neonatal CNS. Mol Cell Neurosci 13: 143–166. 10192772 10.1006/mcne.1999.0738

[15] Nakamura F, Kalb RG, Strittmatter SM (2000) Molecular basis of semaphorin-mediated axon guidance. J Neurobiol 44: 219–229. 10934324 10.1002/1097-4695(200008)44:2<219::aid-neu11>3.0.co;2-w

[16] Kubilus JK, Linsenmayer TF (2010) Developmental guidance of embryonic corneal innervation: roles of Semaphorin3A and Slit2. Dev Biol 344: 172–184. 20471970 10.1016/j.ydbio.2010.04.032 PMC4283142

[17] Lwigale PY, Bronner-Fraser M (2007) Lens-derived Semaphorin3A regulates sensory innervation of the cornea. Dev Biol 306: 750–759. 17499699 10.1016/j.ydbio.2007.04.012

[18] Tanelian DL, Barry MA, Johnston SA, Le T, Smith GM (1997) Semaphorin III can repulse and inhibit adult sensory afferents in vivo. Nat Med 3: 1398–1401. 9396612 10.1038/nm1297-1398

[19] Morishige N, Ko JA, Liu Y, Chikama T, Nishida T (2008) Localization of semaphorin 3A in the rat cornea. Exp Eye Res 86: 669–674. 18308303 10.1016/j.exer.2008.01.012

[20] Kikuchi K, Kishino A, Konishi O, Kumagai K, Hosotani N, et al (2003) In vitro and in vivo characterization of a novel semaphorin 3A inhibitor, SM-216289 or xanthofulvin. J Biol Chem 278: 42985–42991. 12933805 10.1074/jbc.M302395200

[21] Kaneko S, Iwanami A, Nakamura M, Kishino A, Kikuchi K, et al (2006) A selective Sema3A inhibitor enhances regenerative responses and functional recovery of the injured spinal cord. Nat Med 12: 1380–1389. 17099709 10.1038/nm1505

[22] Acevedo LM, Barillas S, Weis SM, Gothert JR, Cheresh DA (2008) Semaphorin 3A suppresses VEGF-mediated angiogenesis yet acts as a vascular permeability factor. Blood 111: 2674–2680. 18180379 10.1182/blood-2007-08-110205 PMC2254547

[23] Stuart PM, Pan F, Plambeck S, Ferguson TA (2003) FasL-Fas interactions regulate neovascularization in the cornea. Invest Ophthalmol Vis Sci 44: 93–98. 12506060 10.1167/iovs.02-0299

[24] Kawakita T, Shimmura S, Hornia A, Higa K, Tseng SC (2008) Stratified epithelial sheets engineered from a single adult murine corneal/limbal progenitor cell. J Cell Mol Med 12: 1303–1316. 18318692 10.1111/j.1582-4934.2008.00297.x PMC3225011

[25] Ko JA, Mizuno Y, Yanai R, Chikama T, Sonoda KH (2010) Expression of semaphorin 3A and its receptors during mouse corneal development. Biochem Biophys Res Commun 403: 305–309. 21075075 10.1016/j.bbrc.2010.11.022

[26] Ko JA, Morishige N, Yanai R, Nishida T (2008) Up-regulation of semaphorin 3A in human corneal fibroblasts by epidermal growth factor released from cocultured human corneal epithelial cells. Biochem Biophys Res Commun 377: 104–108. 18831963 10.1016/j.bbrc.2008.09.085

[27] Morishige N, Ko JA, Morita Y, Nishida T (2010) Expression of semaphorin 3A in the rat corneal epithelium during wound healing. Biochem Biophys Res Commun 395: 451–457. 20331965 10.1016/j.bbrc.2010.03.124

[28] Cao Z, Wu HK, Bruce A, Wollenberg K, Panjwani N (2002) Detection of differentially expressed genes in healing mouse corneas, using cDNA microarrays. Invest Ophthalmol Vis Sci 43: 2897–2904. 12202508

[29] Rosenberg ME, Tervo TM, Immonen IJ, Muller LJ, Gronhagen-Riska C, et al (2000) Corneal structure and sensitivity in type 1 diabetes mellitus. Invest Ophthalmol Vis Sci 41: 2915–2921. 10967045

[30] Gallar J, Tervo TM, Neira W, Holopainen JM, Lamberg ME, et al (2010) Selective changes in human corneal sensation associated with herpes simplex virus keratitis. Invest Ophthalmol Vis Sci 51: 4516–4522. 20375335 10.1167/iovs.10-5225

[31] Halberstadt M, Machens M, Gahlenbek KA, Bohnke M, Garweg JG (2002) The outcome of corneal grafting in patients with stromal keratitis of herpetic and non-herpetic origin. Br J Ophthalmol 86: 646–652. 12034687 10.1136/bjo.86.6.646 PMC1771166

[32] Afonso AA, Monroy D, Stern ME, Feuer WJ, Tseng SC, et al (1999) Correlation of tear fluorescein clearance and Schirmer test scores with ocular irritation symptoms. Ophthalmology 106: 803–810. 10201606 10.1016/S0161-6420(99)90170-7

[33] Chang JH, Javier JA, Chang GY, Oliveira HB, Azar DT (2005) Functional characterization of neostatins, the MMP-derived, enzymatic cleavage products of type XVIII collagen. FEBS Lett 579: 3601–3606. 15978592 10.1016/j.febslet.2005.05.043

[34] Cursiefen C, Masli S, Ng TF, Dana MR, Bornstein P, et al (2004) Roles of thrombospondin-1 and -2 in regulating corneal and iris angiogenesis. Invest Ophthalmol Vis Sci 45: 1117–1124. 15037577 10.1167/iovs.03-0940

[35] Gabison E, Chang JH, Hernandez-Quintela E, Javier J, Lu PC, et al (2004) Anti-angiogenic role of angiostatin during corneal wound healing. Exp Eye Res 78: 579–589. 15106938 10.1016/j.exer.2003.09.005

[36] Maddula S, Davis DK, Burrow MK, Ambati BK (2011) Horizons in therapy for corneal angiogenesis. Ophthalmology 118: 591–599. 21376242 10.1016/j.ophtha.2011.01.041 PMC4107641

[37] Cai D, Shen Y, De Bellard M, Tang S, Filbin MT (1999) Prior exposure to neurotrophins blocks inhibition of axonal regeneration by MAG and myelin via a cAMP-dependent mechanism. Neuron 22: 89–101. 10027292 10.1016/s0896-6273(00)80681-9

[38] Morgenstern DA, Asher RA, Fawcett JW (2002) Chondroitin sulphate proteoglycans in the CNS injury response. Prog Brain Res 137: 313–332. 12440375 10.1016/s0079-6123(02)37024-9

[39] Bradbury EJ, Moon LD, Popat RJ, King VR, Bennett GS, et al (2002) Chondroitinase ABC promotes functional recovery after spinal cord injury. Nature 416: 636–640. 11948352 10.1038/416636a

[40] Li YN, Pinzon-Duarte G, Dattilo M, Claudepierre T, Koch M, et al (2012) The expression and function of netrin-4 in murine ocular tissues. Exp Eye Res 96: 24–35. 22281059 10.1016/j.exer.2012.01.007 PMC3296891

[41] Kaplan N, Fatima A, Peng H, Bryar PJ, Lavker RM, et al (2012) EphA2/Ephrin-A1 signaling complexes restrict corneal epithelial cell migration. Invest Ophthalmol Vis Sci 53: 936–945. 22247486 10.1167/iovs.11-8685 PMC3317430

[42] Kumagai K, Hosotani N, Kikuchi K, Kimura T, Saji I (2003) Xanthofulvin, a novel semaphorin inhibitor produced by a strain of Penicillium. J Antibiot (Tokyo) 56: 610–616. 14513903 10.7164/antibiotics.56.610

[43] Yamauchi Y, Abe K, Mantani A, Hitoshi Y, Suzuki M, et al (1999) A novel transgenic technique that allows specific marking of the neural crest cell lineage in mice. Dev Biol 212: 191–203. 10419695 10.1006/dbio.1999.9323

[44] Kawamoto S, Niwa H, Tashiro F, Sano S, Kondoh G, et al (2000) A novel reporter mouse strain that expresses enhanced green fluorescent protein upon Cre-mediated recombination. FEBS Lett 470: 263–268. 10745079 10.1016/s0014-5793(00)01338-7

